# *Saccharibacteria* (TM7) in saliva and subgingival microbiome as a predictor for gingivitis in individuals with type2 diabetes evaluated by qPCR

**DOI:** 10.3389/fdmed.2025.1550936

**Published:** 2025-04-22

**Authors:** Boy M. Bachtiar, Dicky L. Tahapary, Turmidzi Fath, Citra F. Theodorea, Natalina Haerani, Yuniarti Soeroso, Selvi Nafisa Shahab, Ardy Wildan, Fergie Marie Joe Grizella Runtu, Fatimah Maria Tadjoedin, Dewi Ayuningtyas, Lisa Amir, Endang W. Bachtiar

**Affiliations:** ^1^ Department of Oral Biology and Oral Science Research Center, Faculty of Dentistry, Universitas Indonesia, Jakarta, Indonesia; ^2^ Clinical Research Unit RSCM, Metabolic-Endocrine-Diabetes Division, Department Internal Medicine, Faculty of Medicine, Universitas Indonesia - Ciptomangunkusumo Hospital Metabolic Disorder, Cardiovascular, and Aging Research Cluster, Indonesian Medical Education and Research Institute, Jakarta, Indonesia; ^3^ Department of Periodontology, Faculty of Dentistry, Universitas Indonesia, Jakarta, Indonesia; ^4^ Clinical Research Unit RSCM, Department Microbiology, Faculty of Medicine, Universitas Indonesia - Ciptomangunkusumo Hospital, Jakarta, Indonesia; ^5^ Clinical Research Unit RSCM, Jakarta, Indonesia; ^6^ Metabolic-Endocrine-Diabetes Division, Department of Internal Medicine, FKUI-RSCM, Jakarta, Indonesia

**Keywords:** diabetes, *Saccharibacteria*, *Schaalia odontolytica*, gingivitis, periodontitis

## Abstract

Oral samples are widely used for studying oral microbiome in health and diseases. In this study, saliva and subgingival biofilm (SGB) samples obtained from patients with type2 diabetes (T2DM), without periodontitis (G1 group), with gingivitis (G2 group), and periodontitis (G3 group), were used to compare the abundance of *Saccharibacteria* (TM7), its host's bacteria (*Schaalia odontolytica*), periodontopathogen (Represented by *Fusobacterium nucleatum*), and nitrate-reducing bacteria (represented by *Rothia mucilaginosa*). The gingival crevicular fluid were also used to analyze the transcription levels of interleukin-6 (IL-6) and C-reactive protein (CRP). Healthy individuals' oral samples served as a control, and the targeted bacteria and inflammatory indicators were detected and measured using real-time PCR. The results showed that in either sample, the abundance of TM7 and other targeted bacteria showed a similar profile. Notably, within participants with T2DM, the abundance of TM7 was similar in G1 and G2 groups, but significantly decreased in G3 group. With the exception of the SGB of the G3 group, the relationship between TM7 and its bacterial host was strongly positive across all evaluated samples. Furthermore, CRP had higher transcription levels than IL-6 across the entire group. Despite the fact that the G3 group showed an adverse relationship between TM7 and CRP, patients with T2DM generally showed a positive correlation between TM7 and IL-6/CRP, which was verified by a receiver operating curve.

## 1 Introduction

The human oral cavity has emerged as a model for microbiome investigation to comprehend microbial ecology and functionality because of its accessibility and therapeutic significance (1, 2). A remarkably rich bacterial community resides in the human mouth, yet only about half of these microorganisms are culturable (3). Among the unculturable oral bacteria, are Candidate Phyla Radiation (CPR) bacteria, particularly *Saccharibacteri*a (TM7), have received increasing attention. TM7 have been identified as commensal members of various body sites in healthy humans, including the oral cavity, intestine, skin, and stomach (4, 5).

While some studies suggest the presence of several members of the TM7 phylum as the human oral microbiome was associated with periodontal disease, others indicate their potential to inhibit bone loss and inflammation (6, 7) Therefore, understanding the role of TM7 in oral health is significant because these bacteria may influence the onset and progression of periodontal diseases. Their interaction with other microorganisms, such as periodontopathogens and nitrate-reducing bacteria, could affect microbial balance and inflammation in the oral cavity. Moreover, studying TM7 can provide insights into potential therapeutic targets for preventing or managing oral health issues, particularly in individuals with conditions like type-2 diabetes (T2DM). This metabolic disorder can be brought on by a malfunction in either the action or secretion of insulin, or both (8), and periodontitis has been found as another common complication of diabetes mellitus (9, 10).

In this study, association between TM7, periodontopathogens (represented by *Fusobacterium nucleatum*), and nitrate-reducing bacteria (represented by *Sachaalia odontolytica* and *Rothia mucilaginosa* in saliva and subgingival biofilm (SGB) in individuals with T2DM with and without periodontal diseases is investigated. Despite bacteria, the current study additionally explored host salivary biomarkers to monitor the progression of periodontal disorders. This is because saliva, a biomarker sample of oral fluid, can also be affected by systemic inflammatory and infectious conditions (11, 12).

## 2 Material and methods

### 2.1 Subjects and sampling

Patients were recruited from the Dr. Cipto Mangunkusumo Hospital in Jakarta. Oral samples were collected from all individuals who met the inclusion and exclusion criteria. Only people with type-2 diabetes (T2DM; noninsulin-dependent diabetes), aged 20–40 years, and of the sex 35%–50% male, were comparable across the three groups. These individuals were diagnosed with the disease if their blood glucose level was at least 200 mg/dl 2 h after an oral glucose load, their HbA1c level was at least 6.5%, and their plasma glucose level was at least 200 mg/dl with a typical hyperglycaemic crisis. In addition, each participant fulfilled the subsequent requirements: (1) did not smoke or use non-steroidal anti-inflammatory drugs, and did not use any medications, including hormones or antibiotics, in the six months before the sample collection; (2) had a minimum of 20 teeth and had no overt symptoms of stomatitis or root caries; (3) did not consume any food for at least 1 h prior to sample collection. The following patients were excluded from the study: (1) diagnosed as having an underlying condition other than diabetes, such as hyperthyroidism or cancer, which could have contributed to the development of periodontitis; (2) had undergone scaling as part of periodontal therapy within six months prior to the sample collection; (3) was pregnant or breastfeeding during the study. The participants who were included were then divided into three groups: those with diabetes who did not have periodontal disorders (*n* = 12, Group 1), those who had gingivitis (*n* = 12, Group 2), those who had periodontitis (*n* = 12, Group 3), and control (*n* = 7, neither diabetes or periodontal disease) that served as the qPCR analysis's control.

Gingivitis was diagnosed using the bleeding on probing (BOP) score (13), whereas chronic periodontitis was diagnosed using the guidelines previously described (14). Individuals who were diagnosed with chronic periodontitis had more than four sites with probing depth (PD) ≥4 mm and clinical attachment loss (CAL) ≥2 mm, and at least 30% of the sites had alveolar bone resorption.

The study protocols were approved by the Ethics Committee of the Dr. Cipto Mangunkusumo Hospital (ethical reference number: KET-1203/UN2.F1/ETIK/PPM.00.02/2023), and all participants gave written informed consent prior to participation in the study in accordance with the requirements of the Ethics Committee.

### 2.2 Saliva, subgingival plaque, and gingival crevicular fluid samples collection and DNA extraction

Two milliliters of unstimulated saliva was collected by asking the participants to drool into a sterile 15-ml screw-capped centrifuge tube. Samples for subgingival biofilm (SGB) and gingival crevicular fluid (GCF) analysis were obtained by inserting 3–5 sterile endodontic paper points for 30 s into the sulcus of mesial surface of a premolar or first molar tooth. The paper points were then placed in a 500 µl microcentrifuge filled with phosphate buffer saline (PBS). Saliva and SGB/GCF samples were immediately transported to the lab, where centrifugation was used to separate the GCF and SGB's biofilm (15). Using GENEzol™ reagents (phenol, guanidine isothiocyanate solution) (Geneaid Biotech Ltd, New Taipei City, Taiwan), bacterial DNA was then extracted from saliva and SGB samples in accordance with the company's procedure. The concentration and quality of the DNA obtained were determined using Qubit assay reagents (Thermo Fisher Scientific, Waltham, MA, United States).

### 2.3 DNA amplification

Three separate quantitative real-time PCR (qPCR) assays were performed using specific primers for *F. nucleatum. S. odontolyticus*, and *R. mucilaginosa* using the LightCycler-96 (Roche Inc., Branchburg, NJ, USA). The PCR reaction was performed under the following conditions: an initial denaturation step at 95°C for 5 min, followed by 35 cycles of denaturation at 95°C for 5 s, annealing at 60°C for 15 s, and extension at 72°C for 15 s. A final cooling step of 30 s at 40°C was included to ensure complete DNA synthesis and proper annealing. The relative abundance of each targeted bacteria was determined using the relative calculation of the ΔΔ Ct formula (2^−ΔΔCt^) (15). For the IL-6 and C-reactive protein (CRP), complementary DNA (cDNA) of gingival crevicular RNA was amplified using qPCR (16). Primers listed in Table 1 were used to perform the PCR. The relative expression of the mRNA was determined using the 2^−ΔΔCT^ technique.

**Table 1:** Primers used in this study.

Bacteria	Primer sequences (“5—3”)	References
TM7	CAGTCCAAGTAGAAAAATAC	(35)
	TATGAGTGAAGAATATGA	
*S. odontolyticus*	GCGGATTAATTCGATGCAACGCGA	(36)
	CATTGTAGCATGCGTGAAGCCCAA	
*F. nucleatum*	TCCCAGCAAATGTTGGAAG	(37)
	TTCATCATCAAATTCGTCATAGTCT	
*R. mucilaginosa*	ACACGTGAGTAACCTACCCTT	(38)
	GCAGGTACCGTCAATCTCTC	
16S rRNA	AGAGTTTGATCMTGGCTCAG	(39, 40)
	CGTATTACCGCGGCTGCTGG	
CRP	TGGCCAGACAGACATGTCGAGG	(16)
	AGTGGAGGCACACAGTGAAGGC	
IL-6	ACAGCCACTCACCTCTTCAG	(16)
	CCATCTTTTTCAGCCATCTTT	
GAPDH	TTGGCTACAGCAACAGGGTG	(41)
	GGGGAGATTCAGTGTGGTGG	

### 2.4 Statistical analysis

The analysis was conducted using GraphPad PRISM 9.4 (GraphPad Software, San Diego, CA, USA). The DNA load abundance for each species was calculated separately before the distribution across the four groups of subjects was compared. Data are presented as mean ± standard error (SE), and a *p*-value of <0.05 was considered statistically significant. For comparison between groups, one-way ANOVA was used, while for comparison within groups, the unpaired Student's *t*-test was used. For correlation analysis, Spearman's correlation coefficient (*r*) was calculated, and linear regression was used to generate the line of best fit with 95% confidence intervals. The receiver operating characteristic (ROC) curve analysis was performed to determine the relationship between two independent variables with high sensitivity and specificity.

## 3 Results

### 3.1 Abundance of TM7, *S. odontolyticus*, *F. nucleatum*, and *R. mucilaginosa*

**Figure 1:**
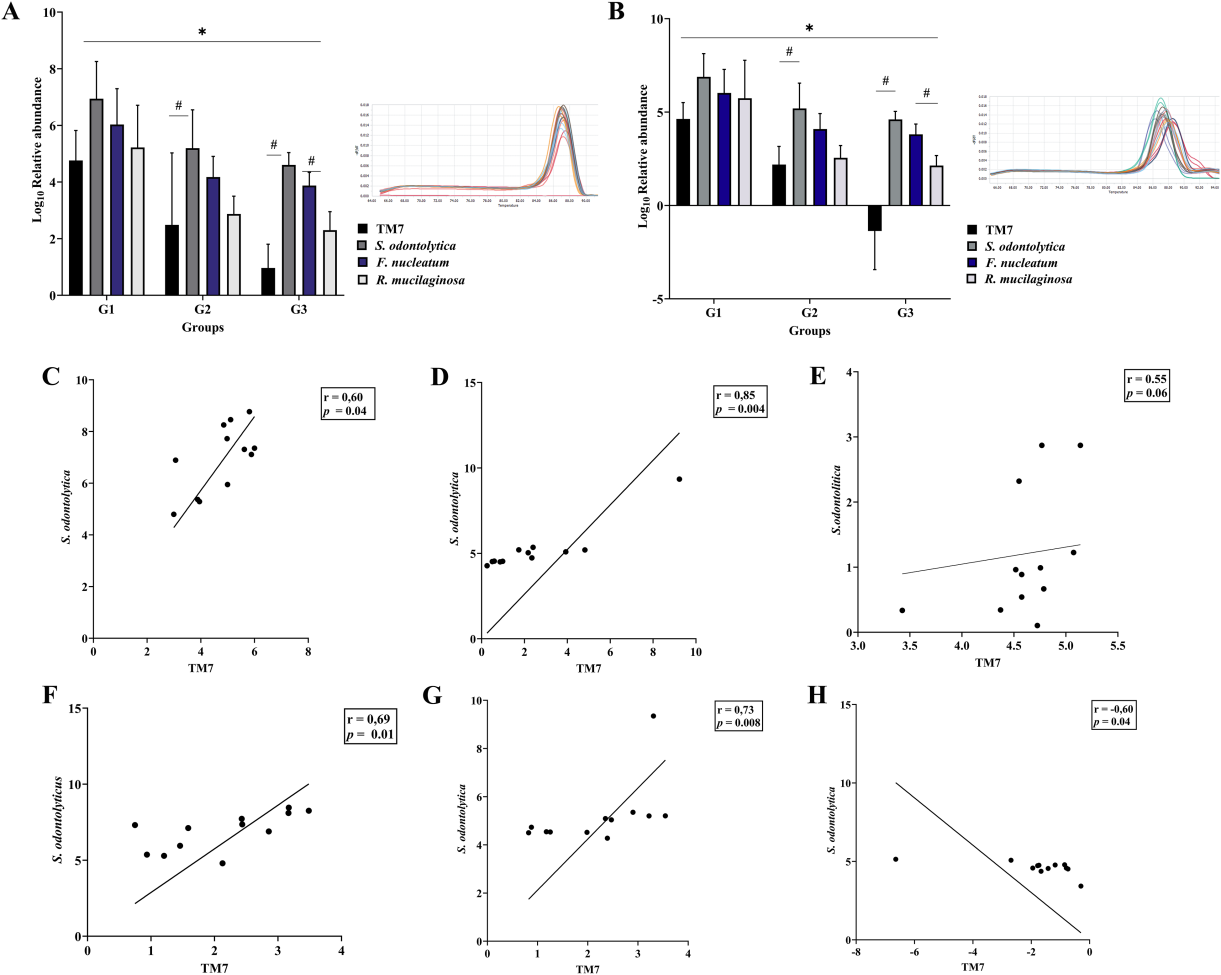
The proportions of TM7, *S. odontolyticus*, *F. nucleatum*, and *R. mucilaginosa*, as well as the relationships between TM7 and its host bacteria. Three groups of T2DM patients (G1, G2, and G3) are compared in the upper panel for the relative abundance of TM7 and the selected bacteria in saliva **(A)** and subgingival plaque **(B)** likewise the melting curves of TM7 and *S. odontolyticus* inserted in A and B, respectively, are displayed. The lower panel shows scatter diagram illustrating the correlation between TM7 and *S. odontolytica* in saliva's patients with T2DM. These findings suggest that the abundance of TM7 and *S. odontolytica* exhibit a substantial positive link, which was observed in the G1 and G2 groups, while the positive association in G3 group was not significant. (*) indicates a significant correlation between groups, while (#) indicates significant correlation within groups. Spearman coefficient correlation (*r*) and *p* value are given.

In both samples, the G1 group had a higher abundance of TM7 and other studied bacteria than the G2 and G3 groups (*p* < 0.05) (Figures 1A,B). When bacterial abundance in the presence of periodontal inflammation (gingivitis and periodontitis) was compared, it was found that the G1 group's bacteria gradually declined in the G2 and G3 groups, both in saliva and in SGB, with the exception of the proportion of TM7, which significantly decreased in SGB.

### 3.2 The relationship between the TM7 proportion and *S. odontolyticus* abundance

The linear regression analysis revealed a positive and significant correlation between the abundance of *Saccharibacteria* and the proportion of *S. odontolyticus* in saliva. The significant strong association was observed in G1 (*r* = 0.60, *p* = 0.04) and G2 (*r* = 0.85, *p* = 0.004) groups, while in G3 group the positive correlation was not significant (*r* = 0.55, *p* = 0.06) (Figures 1C–E), In SGB, the linear positive significant association was observed in both G1 (*r* = 0.69, *p* = 0.001) and G2 (*r* = 0.73, *p* = 0.008) groups, but a negative significant correlation was observed in G3 group (*r* = −0.60, *p* = 0.04) (Figures 1F–H).

### 3.3 The correlation between the TM7 proportion, Il6/CRP transcription levels, and the area under the ROC curve

**Figure 2:**
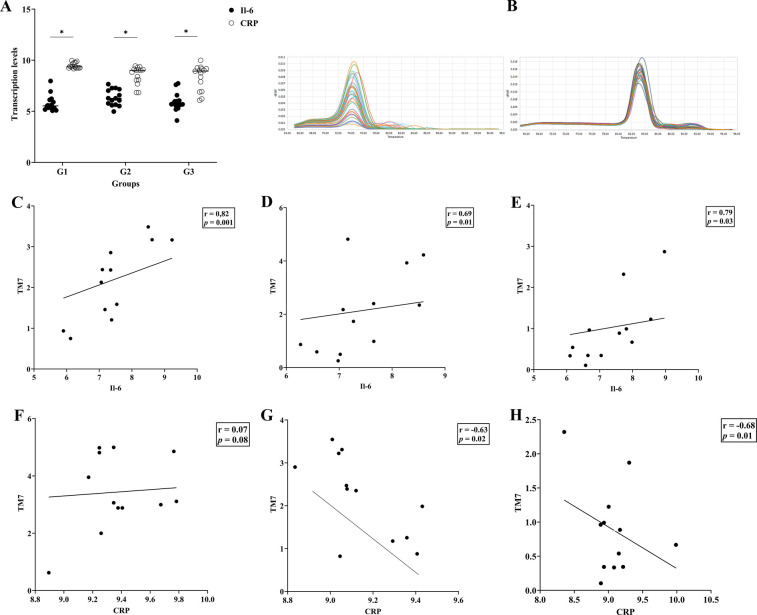
Interleukin-6 (IL-6) and C-reactive protein (CRP) transcription levels and their associations with TM7 in T2DM patients with and without periodontal disease. The upper panel **(A)** shows the transcription levels of the inflammation markers (IL-6 and CRP) in three groups of T2DM patients (G1, G2, and G3). Additionally shown in **(B)** are the melting curves for Il-6 (left) and CRP (right). The relationship between TM7 and Il-6 **(C–E)** and CRP **(F–H)** for each tested group is displayed in the middle and lower panels, respectively. (*) indicates a significant correlation. Spearman correlation coefficient (*r*) and *p* value are indicated.

**Figure 3:**
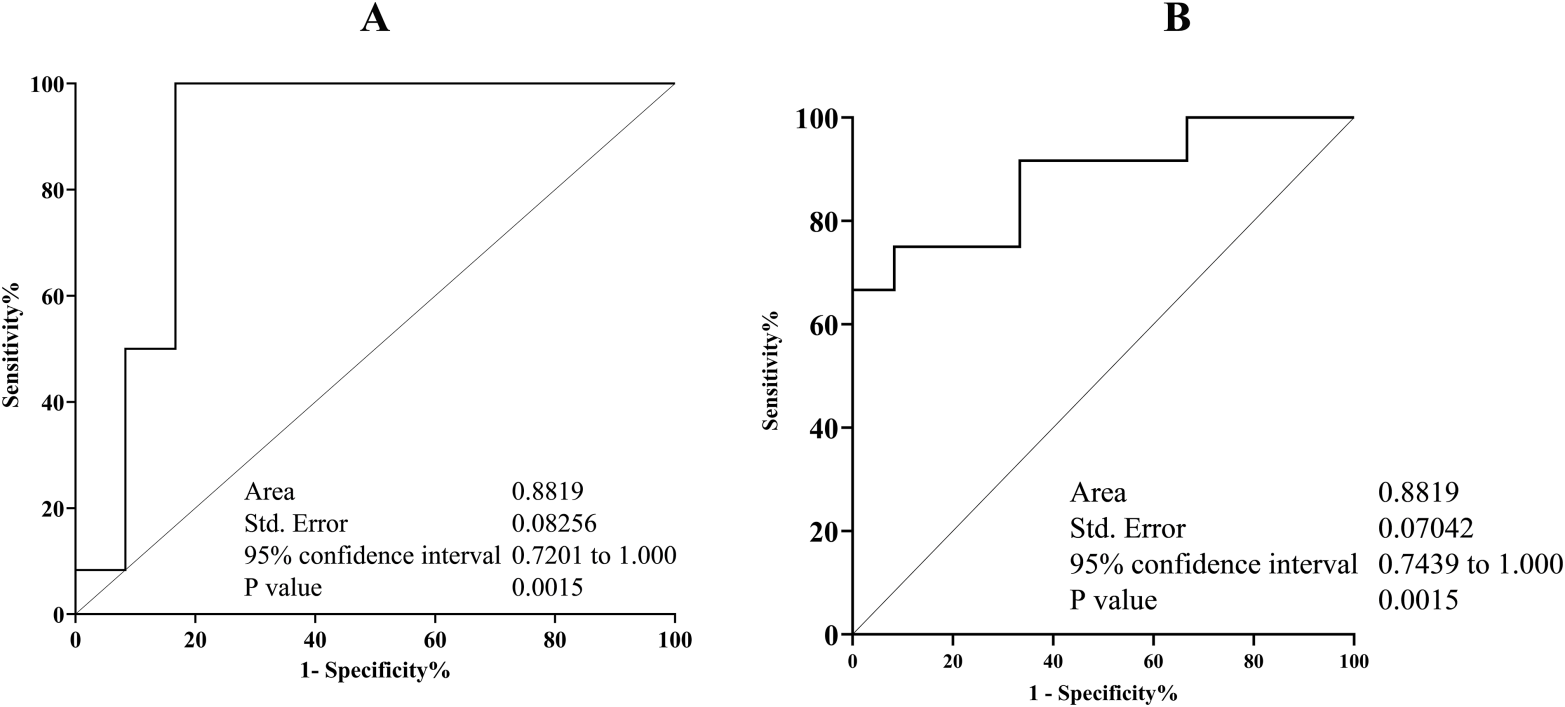
Receiver operating characteristic (ROC) displaying the relationship between TM7 and *S. odontolyticus*/CRP plot and appropriate cut-off point. The combination of TM7 and *S. odontolyticus* abundance in saliva **(A)** and gingival crevicular fluids **(B)** could discriminate the initial inflammation of periodontal diseases in T2DM patient. The area under cover (AUC) and *p* value are indicated.

Figure 2A displays the IL-6 and CRP transcription levels as well as their melting curve resulted from PCR amplification (Figure 2B). Different patient groups had different levels of Il- 6 and CRP mRNA expression in gingival crevicular fluids (GCF). The expression of Il-6 was significantly lower than CRP in each group (*p* < 0.05). The saliva sample in Figures 2C–E demonstrates that there was a strong positive connection between the transcription level of Il-6 and the abundance of TM7 in all groups (G1; *r* = 0.82, *p* = 0.01), (G2; *r* = 0.69, *p* = 0.01), and (G3; *r* = 0.79, *p* = 0.03). In SGB/GCF, G1group showed a weak positive association and not significant, between TM7 levels and the transcription level of Il-6 (*r* = 0.07, *p* *=* 0.08), while a strong significant inverse correlation was observed in G2 (*r* = −0.63, *p* = 0.02) and G3 (*r* = −0.68, *p* = 0.01) groups (Figures 2F–H). Based on the results, we applied ROC curve analysis to evaluate the additional value in order to predict the performance relationship of the interaction between the abundance of TM7 and the transcription levels of CRP (Figure 3A,B). The ROC curve analysis shows that the G2 group demonstrated a consistently statistically significant diagnostic strength in both saliva and GCF, with AUCs of 0.08 (*p* = 0.001).

## 4 Discussion

This study examines the amount of certain oral microbiota in T2DM patients with and without periodontitis, taking into account TM7's impact on periodontal disorders, which are among the most common forms of oral disease (17). Additionally, salivary CRP and IL-6 were included since they can be non-invasive biomarkers of periodontitis and systemic diseases (16).

Certain periodontal bacteria have been linked to type 2 diabetes/T2DM (18). Our study's findings revealed that TM7, its bacterial host (*S. odontolytica*), and the inflammatory markers CRP and Il-6 provide good diagnostic accuracy when determining the degree of periodontal inflammation in individuals with type 2 diabetes. First, we evaluated the number of the selected bacteria (TM7, *S. odontolytoca*, *F. nucleatum*, and *R. mucilaginosa*) considered to be representative of the oral microbiome of individuals with type 2 diabetes who had and did not have periodontal inflammation. To limit the heterogeneity and variability between subjects, the differences in microbiome composition between patients groups were analysed on young patients in specific age group. As qPCR has a high sensitivity for detecting and quantifying bacteria, we used it to investigate the bacterial abundance (19). Our results demonstrated that all of the selected bacteria were present in the saliva and periodontal niche of each individual in the study group (G1, G2, G3, and control). This suggest that the bacteria are present in the mouth as normal flora and that they contribute to the development of periodontal diseases in people with type 2 diabetes. We also observed that the abundance of *F. nucleatum* and *R. mucilaginosa*, as well as the numbers of TM7 and *S. odontolytica*, varied significantly based on the participants' level of periodontal health. This suggests that young adults with type 2 diabetes may acquire periodontal disease as a result of these bacterial compositions.

Type 2 diabetes (T2DM) is a major risk factor for periodontal disease, and individuals with this condition tend to have more severe and aggressive periodontal inflammation (20). This study discovered that in both clinical samples examined (saliva and subgingival biofilm/SGB), the most abundant bacteria in our subjects with T2DM were *S. odontolyticus*, *F. nucleatum*, and *R. mucilaginosa*, while TM7 was the least abundant. We also found that the amount of TM7 in the saliva or SGB of the G1, G2, and G3 groups gradually decreased, which is contrary to a previous result that TM7 abundance increases periodontal inflammation (2, 6, 21, 22). Thus, our results may indicate that in people with type 2 diabetes, reduced TM7 may be related to or influence the onset of periodontal disorders.

Clinical research has shown that TM7 is associated with gingivitis and periodontitis (21), and the bacteria's interaction with *S. odontolyticus* has been revealed to exacerbate the inflammatory processes of periodontal tissue (23, 24). Our findings provided additional insight into how the degree of periodontal disease affects the dynamic interactions between TM7 and its bacterial host. We noticed that the amount of TM7 in saliva was positively correlated with the number of *S. odontolyticus*, but this strong correlation was only observed in gingivitis (G2 group) and periodontal health (G1 group). This suggests that the saliva of diabetics maintains TM7 and its bacterial host. However, there was a reverse correlation in the subgingival plaque of individuals with periodontitis (G3 group). This might suggest that TM7's interactions with its bacterial host may alter the bacterial population's composition in subgingival niche (25), which could affect the development of periodontal disease. Additionally, changes in oxygen tension, nutrition availability, or competitive interactions with other bacteria may result through the inflammatory environment associated with progressive periodontitis (26, 27), which could disrupt the original symbiotic relationship. The results are in contrast to previous studies that showed a rise in TM7 species in people with periodontitis (21, 28). However, our results corroborate earlier research in a mouse model that indicated *S. odontolyticus* can be a pathobiont, with TM7 preventing its pathogenicity (7). Taking this finding into account, more research is required into the specific processes causing the shift in the relationship.

One of the findings in this research was a statistically significant higher prevalence of *F. nucleatum* and *R. mucilaginosa* in saliva and the subgingival microbiota of periodontally healthy people with T2DM. They showed a consistent lower abundance in T2DM subject with periodontal diseases (G2 and G3 groups) compared to G1 group. Based on this finding, we hypothesized that the two species and TM7's decreased abundance in T2DM patients with periodontal disorders are related. Since these bacteria have been linked to nitrite-producing species (29, 30), they deserve further investigation.

The cytokines IL-6 and CRP are essential in the pathophysiology of periodontal diseases (16). They may also be important in metabolic diseases like diabetes (31). Our finding showed, that CRP transcription levels were consistently higher than IL-6 across all T2DM patient groups. This implies that systemic inflammation contributes more to the overall inflammatory load than localized gingival inflammation in early-stage T2DM without overt periodontal disease (16). This result raises the possibility that systemic inflammation, independent of active periodontal inflammation, may worsen inflammatory cascades associated with periodontitis and alter the condition of the periodontal tissues (32). Furthermore, we found a consistently high positive correlation between IL-6 and TM7. Only in the G1 group was a positive correlation observed between TM7 and CRP; in the G2 and G3 groups, there was a significant negative association. Given that CRP is a crucial cytokine that plays a significant role in the progression of various inflammatory diseases (33), we assumed that the decrease in TM7 abundance suggests the active role of systemic inflammation, potentially indicating a shift from gingivitis to chronic inflammation (periodontitis) (34). Both positive and negative significant associations between TM7 and the inflammatory markers (IL-6 and CRP) emphasize the host response in the present study.

Overall, this study finding indicates TM7 has a more complicated role, and our findings provide additional insights regarding the relationship between TM7 and its host bacterium, *S. odontolyticus*. Although the impact of the bacterial relationship on the pathogenesis of periodontal inflammation could not be assessed in this study, the consistence strong positive correlation between TM7 and *S. odontolyticus* in G2 group (T2DM patient with gingivitis) in either sample tested may indicate that these bacteria could be predictive markers for the mild form of periodontal inflammation in T2DM patients. Therefore, to maximize the predictive power of this prediction, the sensitivity and specificity were calculated. Based on ROC curve analysis, the association between TM7 and *S. odontolyticus* and CRP in saliva and gingival crevicular fluids, respectively, demonstrated a stronger predictive value for gingivitis.

## 5 Limitations of the study

This study has several limitations. First, as an observational study, it was challenging to control all possible confounders, which may underestimated the significance of some variables. Moreover, the patient's blood glucose levels were not measured during this study. As a result, data analysis was unable to establish the significance of these variables. Second, the relatively small number of participants in the study limited the ability to determine whether the changes in the oral microbial composition in the saliva and periodontal niche had already occurred at the time oral samples were collected. Lastly, the prevalence of specific bacteria in a patient's oral microbiota is a major determinant in inducing inflammation in periodontal disease, which is a complicated condition. In our analysis, we used relative abundance for the quantity of bacteria in this study rather than use absolute quantification techniques for each targeted species.

## 6 Conclusions

Overall, the findings reveal that individuals with type 2 diabetes mellitus who also have gingivitis or periodontitis may have distinct interactions between TM7 and its host bacterium (*S. odontolyticus*), as well as with periodontopathic and nitrate-reducing bacteria in saliva and the SGB. Periodontal inflammation (gingivitis and periodontitis) did not alter the proportion pattern of the TM7 and its host bacterium, periodontopathogen (*F. nucleatum*), and the representative of healthy species (*R. mucilaginosa*) in patients with T2DM. A potential mechanism by which TM7 may contribute to the development of periodontal diseases in people with type 2 diabetes is suggested by the comparatively low level of TM7 and its relationship with *S. odontolyticus* and CRP in saliva and gingival crevicular fluids, respectively. Therefore, these association appear to be relevant oral fluid biomarkers that might help to identify oral dysbiosis in early periodontal inflammation in our T2DM participants. Our findings may have clinical practice implications, particularly for early detection and treatment of periodontal disease in T2DM patients. Additionally, in the context of periodontal disease in people with T2DM, the inhibition of *S. odontolyticus* by TM7 provides a potential perspective on the function of certain bacteria in oral health and disease. These relationships and their significance for clinical procedures may become clearer with further investigation.

## Data Availability

The original contributions presented in the study are included in the article/Supplementary Material, further inquiries can be directed to the corresponding author.
